# ﻿*Curcumamaxwellii* and *C.rubroaurantiaca* (Zingiberaceae, Zingiberoideae), two new species from Thailand

**DOI:** 10.3897/phytokeys.235.111400

**Published:** 2023-11-20

**Authors:** Jana Leong-Škorničková, Sutthinut Soonthornkalump, Anders Jan Lindström, Sira Niwesrat, Sarah Qing Lim, Piyakaset Suksathan

**Affiliations:** 1 Singapore Botanic Gardens, National Parks Board, 1 Cluny Road, 259569 Singapore, Singapore Singapore Botanic Gardens Singapore Singapore; 2 Department of Biological Sciences, National University of Singapore, 16 Science Drive 4, Singapore 117558, Singapore National University of Singapore Singapore Singapore; 3 Department of Agriculture and Resources, Faculty of Natural Resources and Agro-Industry, Kasetsart University Chalermphrakiat Sakon Nakhon Province Campus, Chiang Khruea, Mueang Sakon Nakhon, Sakon Nakhon, 47000, Thailand Kasetsart University Chalermphrakiat Sakon Nakhon Province Campus Sakon Nakhon Thailand; 4 Nong Nooch Tropical Botanic Garden 34/1, Najomtien, Sattahip, Chonburi, 20250, Thailand Nong Nooch Tropical Botanic Garden Sattahip Thailand; 5 31/360 Moo 17, Bueng Kham Phroi, Lumlukka, Pathumthani 12150, Thailand Unaffiliated Pathumthani Thailand; 6 Queen Sirikit Botanic Garden, Mae Rim, Chiang Mai 50180, Thailand Queen Sirikit Botanic Garden Chiang Mai Thailand

**Keywords:** *Curcumaflammea*, *Curcumalindstromii*, *Curcumarhomba*, gingers, subgenus *Ecomatae*, Least Concern, Zingibereae

## Abstract

*Curcumamaxwellii***sp. nov.** and *Curcumarubroaurantiaca***sp. nov.** (Zingiberaceae, Zingiberoideae, Zingibereae), two new red-orange-flowered species from Thailand, are described. They are compared to the morphologically closest species from the Curcumasubgen.Ecomatae and detailed descriptions, colour plates and information on their distribution, ecology, phenology and uses are provided. Preliminary IUCN conservation assessments for both of these species are proposed as Least Concern

## ﻿Introduction

*Curcuma* L. (Zingiberaceae, Zingibereae) is one of the largest ginger genera widely distributed in South and Southeast Asia and South China, with a few species extending to northern Australia and the South Pacific (Záveská et al. 2012). The number of *Curcuma* species have been steadily rising in past 30 years from about 80 to the current estimate of more than 150 species (Leong-Škorničková et al. 2022). The genus is economically important with turmeric (*Curcumalonga* L.) being perhaps the best-known example, but very many other species are used as spices, condiments, vegetables, medicinal plants and several species are a prominent part of the tropical horticultural industry. The introduction about the genus and its subgenera was given in our recent works (Leong-Škorničková et al. 2015, 2020, 2021) and is, therefore, not repeated here.

Over 65 species from all three subgenera are present in Thailand (Leong-Škorničková et al. 2021), of which more than 20 were described in the last 10 years (e.g. Maknoi et al. (2011, 2019); Chen et al. (2015); Leong-Škorničková et al. (2017, 2020, 2021, 2022); Boonma and Saensouk (2019); Soonthornkalump et al. (2020, 2021, 2022); Saensouk et al. (2021); Rakarcha et al. (2022); Ruchisansakun and Jenjittikul (2023)).

While working on the *Curcuma* account for the Flora of Thailand, we have realised that the herbarium material misidentified as *C.rhomba* Mood & K.Larsen or *Curcumastenochila* Gagnep. in Thailand is heterogeneous. Although most material had similar shape of leaf blades and inflorescences and the flowers were mostly described as orange with red corolla lobes, the geographically distinct clusters, differences in plant indumentum and evidence from an existing photographic material confirmed the need to re-collect living flowering material from different parts of Thailand to make further conclusions on the taxonomic treatment of the taxa involved. Our work on this complex of species with bright orange flowers with red corolla lobes already led to description of *Curcumalindstromii* from Chanthaburi and designating the lectotype of *C.stenochila* (Leong-Škorničková et al. 2022). Additional targeted fieldwork was done in 2023 in the following Provinces: Chiang Rai, Sakon Nakhon, Loei, Chayaphum and Phetchabun. The newly-re-collected material confirmed our suspicion that additional two taxa should be recognised and these are, therefore, described below as *Curcumamaxwellii* and *C.rubroaurantiaca*.

Although it remains unclear if *Curcumastenochila* occurs in Thailand at all, it is clear that both of the two species described here are sufficiently distinct from it as elaborated in notes under each of the two species.

## ﻿Material and methods

The description of these new species is based on measurements from living flowering material and examination of herbarium specimens including flowers preserved in spirit. All extant herbarium material of *Curcuma* was examined at AAU, BK, BKF, BM, CMU, E, K, L, P, PSU, QBG and SING. The style of description follows the recent works of Leong-Škorničková et al. (2013, 2014, 2017, 2020, 2021, 2022). The general plant terminology follows Beentje (2016). The preliminary conservation assessments are based on the guidelines of the IUCN (2022).

## ﻿Taxonomic treatment

### ﻿ Curcuma (Ecomatae) maxwellii

Taxon classificationPlantaeZingiberalesZingiberaceae

Škorničk. & Suksathan sp. nov.

7E35DEEE-C8CF-57DA-A366-179B377FF6EE

urn:lsid:ipni.org:names:77331206-1

Figs 1 , 2 , 3

#### Diagnosis.

Similar to *Curcumarhomba* Mood & K.Larsen in general habit and flower colour, but differs by bracts green to green with slight reddish tinge, puberulent on both sides (versus solid dark red glossy bracts, glabrous on both sides), bracteoles present (vs. bracteoles absent), calyx puberulent throughout (vs. calyx glabrous, except few hairs on teeth), anther with 2–2.5 mm long narrowly conical spurs (vs. ca. 1 mm short broadly conical spurs with blunt apices).

#### Type.

**Figure 1.**
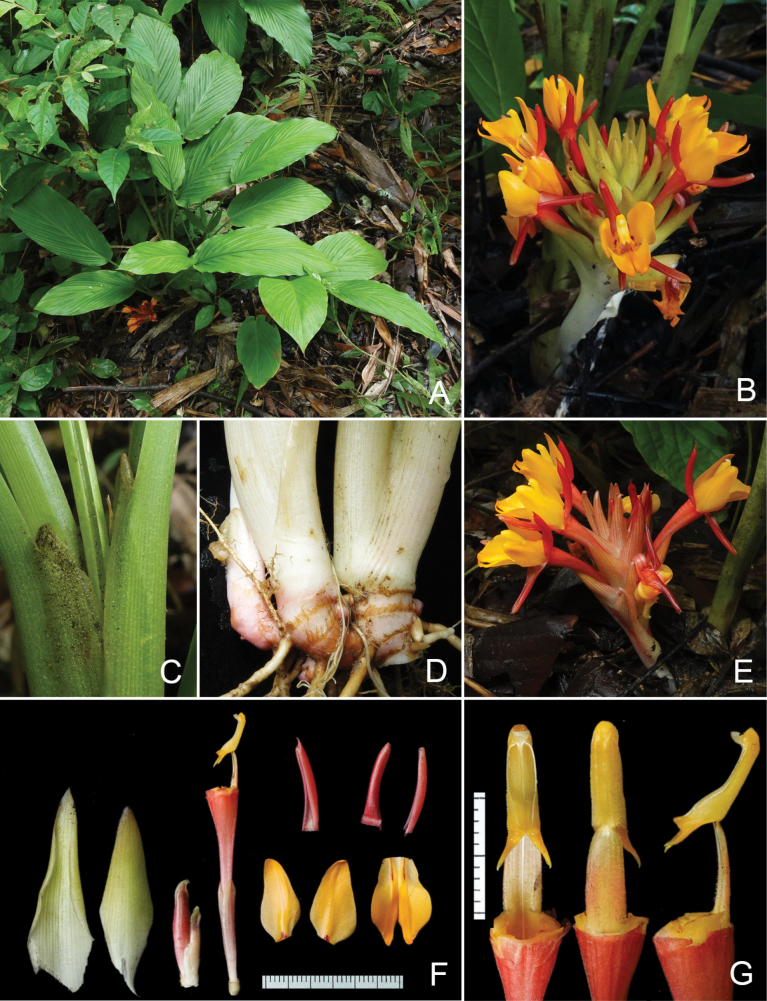
*Curcumamaxwellii* Škorničk. & Suksathan at the type locality **A** habit **B** inflorescence (top view) with detail of flower in front view **C** detail of leaf sheaths and ligules **D** rhizome **E** inflorescence with detail of flower in side view **F** two fertile bracts, three flower buds from the same cincinnus as the dissected flower, dissected flower (from left: floral tube with ovary, calyx and stamen attached, upper row dorsal and lateral corolla lobes, lower row lateral staminodes and labellum **G** stamen still attached to floral tube from front, back and side view. All from the type collection, *Suksathan et al. JLS-4210*. Photographed by Jana Leong-Škorničková.

Thailand, Chiang Rai Province, Chiang Khong District, Rim Khong Subdistrict, 519 m a.s.l., 3 August 2023, *Suksathan et al. JLS-4210* (***Holotype***: QBG! (including flowers in spirit as part of a single preparation); ***Isotypes***: BKF!, E!, P!, SING! (BKF! & SING! including flowers in spirit as part of a single preparation)) Fig. 1.

#### Description.

Rhizomatous herb to 0.8 cm tall; **rhizome** ovoid, ca. 1–1.5 by 0.8–2 cm, with occasional thin branches ca. 4–5 mm diam., brown externally, yellow internally, aromatic with bitter smell; **root tubers** elliptic, ca. 2.5 by 1.1 cm, light brown externally, pure white internally with translucent white centre. **Leafy shoot** 95 cm long with 3–6 leaves when flowering; pseudostem 9–30 cm long, composed of leaf sheaths; **bladeless sheaths** decayed at anthesis; **leaf sheaths** white-green or with pale pink tinge at base turning green distally, glabrous, but pubescent towards the margins; ligule 4–6 mm long, bilobed, lobes round, hyaline, greenish-white, semi-translucent green, hairy; **petiole** 4.5–26 cm long (petiole of first leaf shortest, innermost leaves longer), canaliculate, green, glabrous; **leaf blade** elliptic to elliptic-ovate, 16–44 × 6.5–14 cm, prominently plicate, adaxially bright green, shortly hairy along main veins, abaxially lighter green, glabrous, mid-rib glabrous, green base cordate, apex acuminate, tip ca. 15–20 mm long, pubescent. **Inflorescence** central (often breaking through the pseudostem), many-flowered; **peduncle** 5–12 cm long, to 8 mm diam., white or with reddish tinge; **thyrse** 5–7.5 cm long, 4–6 cm diam. in the middle, without coma; **fertile bracts** 15–34 per inflorescence, 4–4.5 × 1.8–2.7 cm (larger at the base of the inflorescence), ovate to narrowly ovate, smaller at the apex, light green, sometimes with light reddish tinge throughout the bract, puberulent on both sides (slightly less so on the inside) connate in the lower 1/3; enclosing **cincinni** with 4 flowers at the base of the inflorescence, the number of flowers per bract gradually decreasing upwards; **bracteoles** small ca. 1–2 × 0.5–1 mm (outer ones larger, inner ones gradually smaller or totally absent), hyaline, translucent white, glabrous. **Flowers** 6–7 cm, much exserted from the bracts; **calyx** to 22 mm long, 3-toothed, unilaterally split 8–10 mm, semi-translucent white with pink tinge, distally cream to greenish, puberulent; **floral tube** ca. 4.5 cm long, externally pink at the base, gradually redder distally, pubescent, internally light orange, glabrous in basal and distal 1/3, pubescent in middle 1/3, with dorsally placed groove holding the style; **dorsal corolla lobe** 19–21 × 8–10 mm, triangular ovate, with sides rolled inwards, red outside, light orange inside, glabrous on both sides, apex mucronate, mucro ca. 2 mm; **lateral corolla lobes** 19–20 × 7–8 mm, narrowly triangular ovate with sides rolled inwards, glabrous, red on outside, light orange on inside; **labellum** ca. 20 mm long, 5–7 mm broad at basal 5 mm, then broadly ovate, 15–16 mm at widest point, apex bifid with an incision to 7 mm long, labellum orange with darker median bordered by maroon line at basal 4–5 mm; **lateral staminodes** 16–20 × 8–11 mm, narrowly ovate to bluntly rhomboid, orange with small triangular maroon patch at base (ca. 2 mm), glabrous on both sides. **Stamen** 16–18 mm long; **filament** 7–8 mm long, 3.5 mm broad at base, 2 mm broad at apex (the point of attachment to the connective), orange with reddish tinge dorsally, dorsally covered with glandular hairs; **anther** 13–14 mm long, spurred, connective orange, densely covered with short glandular hairs; **anther spurs** 2–2.5 mm long, narrowly triangular with sharp tips pointing outwards; **anther crest** thick, rounded, ca. 1 mm long and ca. 1.5 mm broad at base, orange; **anther thecae** 8–9 mm, forming narrowly obovate shape, dehiscing along their entire length, pollen white. **Epigynous glands** 2, ca. 3 mm long, ca. 0.6 mm diam., cream white. **Style** thin, white, glabrous, placed in a groove in dorsal side of floral tube; stigma ca. 1 mm long, 1 mm wide, white, ostiole ciliate, facing upwards. **Ovary** 2–3 × 2 mm, trilocular, densely hairy, hairs ca. 1 mm long. **Fruits** subglobose, ca. 10 mm in diam. (almost ripe), cream white with very slight pink tinge, pubescent; **seeds** few per capsule (6–10), ca. 4 mm long (almost ripe), light brown, enclosed in semi-translucent white laciniate aril.

#### Habitat and phenology.

Growing in semi-shade to shaded moist area, near streams, in mixed deciduous forest or primary evergreen hardwood forest, at 400–900 m a.s.l., on granite bedrock. The species flowers from June till September, with fruiting presumably extending into November.

#### Distribution.

Only known from Chiang Mai and Chiang Rai Provinces, N Thailand.

#### Eponymy.

**Figure 2.**
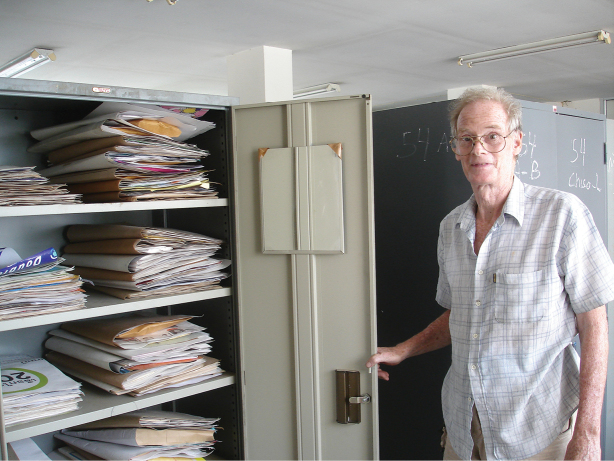
James Franklin Maxwell in CMU Herbarium in 2013. Photographed by J. Leong-Škorničková.

We name this species after our late colleague and remarkable botanist James Franklin Maxwell (1945–2015), also known simply as Max, who collected this species in 1992 (Fig. 2). With more than 32,000 high quality collections, rich in flowers and/or fruits and carefully prepared with many duplicates and mostly with labels that contain much information, Max ranks amongst the best collectors of Thai plants (van Welzen 2023).

#### Vernacular name and uses.

As the vernacular name Wan Pet Ma (ว่านเพชรม้า) is used on several orange-flowered species with red corolla lobes including this species, we propose to use Wan Pet Ma Lanna (ว่านเพชรม้าล้านนา) for this species. Based on the information from the local herbal specialist of the Hmong community, this species, which is locally abundant, has no medicinal uses and only has potential as an ornamental plant.

#### Provisional IUCN conservation assessment.

During our extensive revision of all Thai *Curcuma* material in numerous herbaria (as listed in the Introduction), we have found an additional three herbarium collections, which could be confidently assigned to this species. We predict that the main threats to this species might include excessive collection from the habitat for horticultural purposes and trade, as well as conversion of unprotected areas into agricultural lands. However, the species tend to be locally abundant and at least one of the locations (Lam Nam Kok National Park, Khun Korn Waterfall) is in the legally-protected area. We, therefore, propose to treat this species as Least Concern (LC).

#### Specimens examined.

***Paratypes***: Thailand, Chiang Mai Province, along the road Fang to Chiang Mai; 27 July 1968; *Larsen, K., Santisuk, T. & Warncke, E. 2766*; AAU, BKF; Chiang Rai Province, Mueng District, Koon Gohn Falls [Khun Kon Waterfall], 900 m a.s.l., 20 August 1992, *Maxwell, J.F. 92-440* (AAU, KUN, CMU); Khun Korn Fall [Khun Kon Waterfall], 680 m a.s.l., 22 June 2002, *Chamchumroon, V., Suphuntee, N., Koonkhunthod, N., Ngernsaengsaruay, C. & Tetsana, N. 1601* (BKF, 2 sheets); Doi Tung, 26 Sep 1967, *Iwatsuki, K., Fukoka, N., Hutoh, M. & Chaiglom, D. 13271* (BKF).

#### Notes.

**Figure 3.**
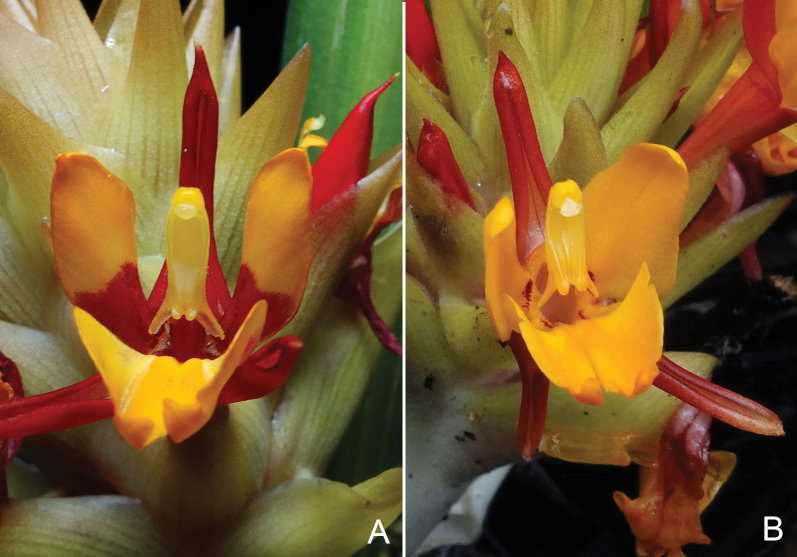
Comparison of flower in front view of **A***Curcumabicolor* and **B***C.maxwellii*. Photographed by Jana Leong-Škorničková.

As already pointed out by Lưu et al. (2017), all the material seen labelled as *C.rhomba* from Thailand in Mood and Larsen (2001) is distinct from the material from southern Vietnam and, in fact, represents several species. The specimen *Larsen & al. 2766* represents *C.maxwellii* and is cited here amongst the paratypes. For this reason, we have compared *Curcumamaxwellii* to *C.rhomba* in the diagnosis. In northern Thailand, *C.maxwellii* might be confused with *Curcumabicolor*, which also has red-orange flowers and is also known to occur in Chiang Mai and Mae Hong Son Provinces. The two species are easy to recognise when flowering as the flowers of *Curcumabicolor* are much more open and the basal half of the staminodes is dark red (Fig. 3).

Outside of Thailand, *Curcumamaxwellii* is also similar to *Curcumaflammea* Škorničk. described from Laos, by general habit and shape of the inflorescence, but differs by adaxially glabrous leaf blades (vs. shortly puberulent), bracts light green with more or less reddish tinge (vs. bracts white, pink to dark red), labellum without prominent basal claw, orange throughout with two thin red lines bordering median band at base (vs. prominently violin-shaped labellum with a prominent broad claw, bright orange with rich red shading and ornamentation), staminodes orange with a small maroon triangular spot at base (vs. staminode mostly bright red with distal part orange), anther with 2–2.5 mm long spurs, not producing mucilage (vs. 3–4 mm long, producing a mucilage in *C.flammea*).

*Curcumamaxwellii* is distinct from *C.stenochila* by the shape of the labellum, which has a narrow basal claw in *C.stenochila*, similar to that of *C.lindstromii*.

### ﻿ Curcuma (Ecomatae) rubroaurantiaca

Taxon classificationPlantaeZingiberalesZingiberaceae

Škorničk. & Soonthornk. sp. nov.

4416C298-CADF-5F1E-8BFB-B10AED7993D2

urn:lsid:ipni.org:names:77331207-1

Fig. 4

#### Diagnosis.

Similar to *Curcumamaxwellii* by general habit and flower colour, but differs by leaf blades abaxially densely puberulent (vs. glabrous), inflorescence composed of up to 14 fertile bracts (vs. inflorescences composed of 15–34 bracts), bracteoles absent (vs. small bracteoles present), larger stamen 19–22 mm long (vs. 16–18 mm long), anther 16–17 mm long with flattened spurs, prominent anther crest 2–3 mm long with central longitudinal groove and anther thecae forming narrowly rhomboid shape (vs. anther 13–14 mm long with conical spurs, thick anther crest ca. 1 mm long without central longitudinal groove and anther thecae forming narrowly obovate shape).

#### Type.

**Figure 4.**
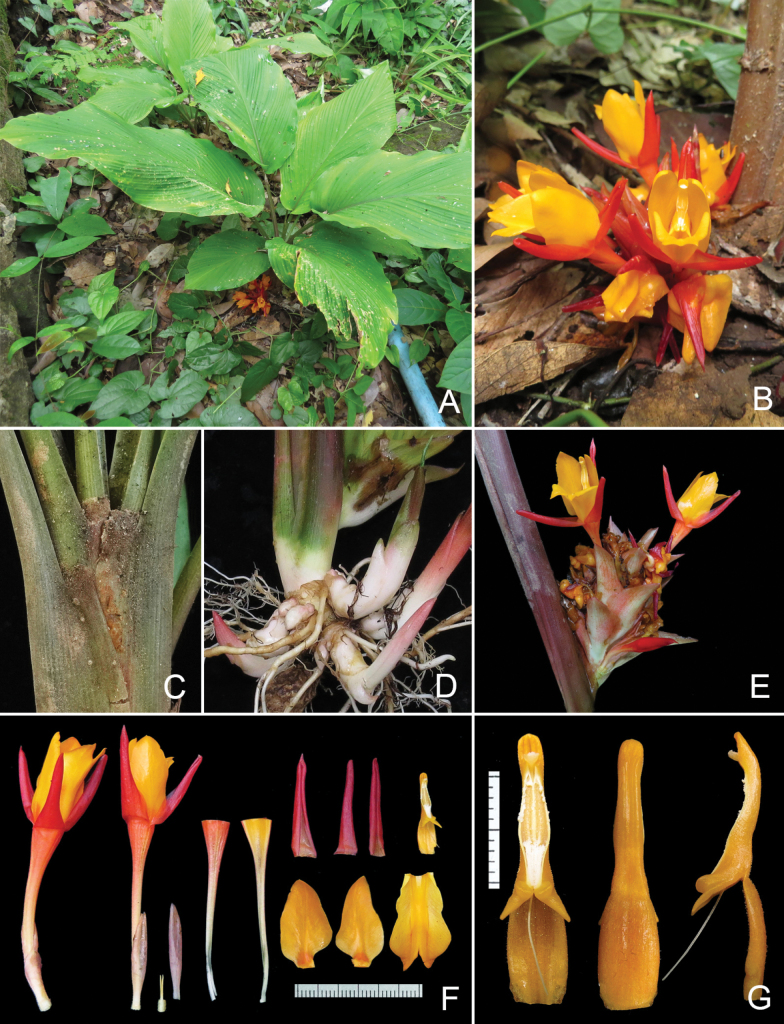
*Curcumarubroaurantiaca* Škorničk. & Soonthornk. at the type locality **A** habit **B** inflorescence with detail of flower in front view **C** detail of leaf sheaths and ligules **D** rhizome **E** inflorescence with detail of flower in side view **F** two flowers and dissected flower (from left: ovary with epigynous glands, calyx, floral tube (dissected longitudinally), upper row dorsal and lateral corolla lobes and stamen, lower row lateral staminodes and labellum **G** stamen from front, back and side view. All from the type collection, *Soonthornkalump Sutt-242*. Photographed by Sutthinut Soonthornkalump.

Thailand, Sakon Nakhon Province, Phanna Nikhom District, Na Hua Bo subdistrict, ca. 200 m a.s.l., 15 July 2023, *Soonthornkalump Sutt-242* (***Holotype***: BKF! (including flowers in spirit as part of a single preparation); ***Isotype***: SING! (including flowers in spirit as part of a single preparation)). Fig. 4.

#### Description.

Rhizomatous herb to 0.6 m tall. **Rhizome** branched, main rhizome ovoid to obvoid, 1.5–2 × 1–2 cm, branches 1–4 cm long, up to 1.2 cm diam., cream-white to ochraceous externally, cream-white internally, slightly aromatic; **root tubers** globose to fusiform 2–4 × 1–2 cm. **Leafy shoot** with 2–5 leaves when flowering; pseudostem to ca. 20 cm long, composed of leaf sheaths; **bladeless sheaths** ca. 3–4, cream-white at base, tinged with rich brownish-red to red distally, fully decayed at anthesis; **leaf sheaths** brownish-red at base, gradually more green with reddish tinge, densely puberulent to puberulent; **ligule** 3–4 mm long, bilobed, lobes rounded to obtuse, brownish-red, densely puberulent; **petiole** (11–)18–30(–45) cm long (petiole of first leaf shortest, innermost leaves longer), canaliculate, green with brownish-red tinge (rarely plain green in distal part), puberulent to densely puberulent, less so in the groove; **leaf blade** broadly elliptic to elliptic-ovate, (12.5–)19–40 × (5–)9–17 cm, prominently plicate, adaxially green, pubescent on main raised veins, near mid-rib and margins, sometimes throughout entire leaf blade, abaxially lighter green, densely puberulent, mid-rib green, puberulent on both sides, base cordate, often slightly oblique, apex acute to acuminate. **Inflorescence** central, breaking through the pseudostem, many-flowered; **peduncle** to 7 mm diam., embedded within pseudostem; **thyrse** 4–8.5 cm long, 3–6 cm diam. in the middle, without coma; **fertile bracts** to 14 per inflorescence, 2–5 × 1–3.6 cm (largest at the base of the inflorescence), ovate to trullate with acute apex, gradually smaller and more ovate towards the apex, margin curved inwards, various colours from white to bright red, shortly puberulent to pubescent on both sides (longer, but sparser hair on the inside), connate in the lower 1/3 to 1/4, enclosing **cincinnus** with 4 flowers at the base of the inflorescence, the number of flowers per bract gradually decreasing to 2 or 1 upwards; bracteoles absent. **Flowers** (5–)6–7.4 cm long, much exserted from the bracts; **calyx** to 24 mm long, 3-toothed, unilaterally split 8–12 mm, semi-translucent with pink-red tinge, pubescent; **floral tube** (30–)40–48 mm long, externally white to pale yellow at the base, gradually with red tinge distally, sparsely shortly pubescent in the middle third, otherwise glabrous, internally pale yellow at the base, bright yellowish-orange distally, mostly glabrous, but pubescent at the point where the tube widens, with dorsally placed groove holding the style; **dorsal corolla lobe** 20–27 × 6–8 mm, triangular elliptic-oblong, with sides rolled inwards, red outside, light red to pale orange inside, glabrous on both sides, apex mucronate, mucro 2–3 mm, with a few sparse hairs; **lateral corolla lobes** 22–25 × 5–8 mm, triangular elliptic with hooded blunt apex, glabrous, red outside, light red to pale orange inside; **labellum** (20–)24–26 × 13–17 mm, 7–9 mm broad at basal 1/3, then bluntly rhomboid in distal 2/3, 13–17 mm at widest point, apex bifid with an incision 5–8 mm long, yellowish-orange, median band thick with central groove, darker orange, sides of the lamina yellow-orange, glabrous and shiny on both sides; **lateral staminodes** 20–22 × 11–13 mm, unequally bluntly rhomboid, bright orange, with pale reddish tinge at base, glabrous and somewhat shiny on both surfaces. **Stamen** ca. 19–22 mm long; **filament** 7–10 mm long, 3.5–4 mm broad at base, 1.5–2 mm broad at apex (the point of attachment to the connective), orange, sparsely covered with glandular hairs; **anther** 16–17 mm long, spurred, connective yellowish-orange, densely covered with short glandular hairs (especially dorsally, less so on the sides); **anther spurs** 2–3 mm long, triangular and flattened with sharp tips pointing outwards; **anther crest** thick ovate to oblong with rounded apex and central longitudinal groove, 1.5–3 mm long and 1.8–2 mm at base, darker orange; **anther thecae** 9–11 mm long, forming very narrowly rhomboid shape, dehiscing along their whole length, connective tissue at the base of the thecae forming blunt knob protruding forward; pollen white. **Epigynous glands** 2, 6–9 mm long, 0.5–1 mm in diam., apex acute, cream at base, yellowish distally. **Style** thin, white, glabrous, held in groove in dorsal side of floral tube; stigma ca. 1 mm long, 1–1.5 mm wide, cream to yellowish, ostiole ciliate, facing upwards. **Ovary** 2.5–3.5 × 2–2.5 mm, trilocular, cream, pubescent, hairs ca. 1 mm long. **Fruit** and **seeds** not seen.

#### Habitat and phenology.

Growing in semi-shade and edges of evergreen forest mixed with bamboo, in moist places, at 200–800 m a.s.l., on sandstone as well as limestone bedrocks. The species flowers from July till September, with fruiting presumably extending into November.

#### Distribution.

Known to occur in Loei, Sakon Nakhon, (NE Thailand), Chayaphum (E Thailand) and Phetchabun Provinces (N Thailand).

#### Etymology.

The specific epithet refers to its bright red and orange flowers.

#### Vernacular name and uses.

Similarly to the previous species, the vernacular name Wan Pet Ma (ว่านเพชรม้า) is used also on this species; we, therefore, propose refining the vernacular name to Wan Pet Ma Isan (ว่านเพชรม้าอีสาน) for this species. No uses were reported, but the species has a good potential as an ornamental plant.

#### Provisional IUCN conservation assessment.

In addition to our collection, we have found an additional two herbarium collections from Loei Province (Phu Luang), two collections from Chayaphum Province (Chulabhorn Dam and Phu Kiew) and two specimens from Petchabun, which could be confidently assigned to this species. Most of these areas are in National Parks under legal protection. The species was also sighted by us in an additional two legally-protected areas, namely Phu Pha Lom Forest Park (Loei Province) and Phu Phan National Park (Sakon Nakhon Province). We, therefore, propose to treat this species as Least Concern (LC).

#### Specimens examined.

***Paratypes***: Thailand, Loei Province, Phu Luang, s.d., *Bunchuai, K. s.n.* (BKF); Phu Luang, 1 September 1966, *Phusomsaeng, S. 26* (BKF); Mueang District, Wang Kan subdistrict, 13 October 2022, *Soonthornkalump, S. Sutt 243* (QBG, including flowers in spirit as part of a single specimen).

#### Notes.

Amongst the orange-red flowered species from this alliance in Thailand, this species is easy to recognise by its anther with well-developed and sometimes slightly recurved anther crest (observed, in particular, in populations in Loei), and thecae forming very narrowly rhomboid shape. This species can be recognised from *C.stenochila* by densely puberulent leaf blades and by the shape of the labellum, which does not have a narrow basal claw as in *C.stenochila*.

### ﻿Key to red-orange flowered species of Curcumasubgen.Ecomatae occurring in Thailand

**Table:** 

1	Lateral staminodes dark red in basal half	***C.bicolor***
–	Lateral staminodes orange throughout, with small red to dark maroon patch either at the base or the tip	**2**
2	Labellum with narrow basal claw and dark maroon almost black tips	***C.lindstromii***
–	Labellum with broad or no basal claw, tips of the same colour (orange) as the rest of the labellum	**3**
3	Leaf blades densely puberulent abaxially, anther with prominent anther crest 2–3 mm long with central longitudinal groove and anther thecae forming narrowly rhomboid shape	***C.rubroaurantiaca***
–	Leaf blades glabrous abaxially, anther with thick anther crest ca. 1 mm long without central longitudinal groove and anther thecae forming narrowly obovate shape	***C.maxwellii***

## Supplementary Material

XML Treatment for Curcuma (Ecomatae) maxwellii

XML Treatment for Curcuma (Ecomatae) rubroaurantiaca

## ﻿Additional information

### Author ORCIDs

Jana Leong-Škorničková https://orcid.org/0000-0003-4490-3490

Sutthinut Soonthornkalump https://orcid.org/0000-0003-4656-578X

Anders Jan Lindström https://orcid.org/0000-0003-0419-9136

Sira Niwesrat https://orcid.org/0009-0003-2707-6785

Piyakaset Suksathan https://orcid.org/0000-0003-4772-4914

### Data availability

All of the data that support the findings of this study are available in the main text.
